# Rotary Friction Welding of Polyetheretherketone Biopolymer Rods Using Variable Rotational Speed

**DOI:** 10.3390/polym15204077

**Published:** 2023-10-13

**Authors:** Chil-Chyuan Kuo, Hua-Xhin Liang, Song-Hua Huang, Shih-Feng Tseng

**Affiliations:** 1 Department of Mechanical Engineering, Ming Chi University of Technology, No. 84, Gungjuan Road, New Taipei City 24301, Taiwan; 2 Research Center for Intelligent Medical Devices, Ming Chi University of Technology, No. 84, Gungjuan Road, New Taipei City 24301, Taiwan; 3 Department of Mechanical Engineering, Chang Gung University, No. 259, Wenhua 1st Road, Guishan District, Taoyuan City 33302, Taiwan; 4 Center for Reliability Engineering, Ming Chi University of Technology, No. 84, Gungjuan Road, Taishan District, New Taipei City 24301, Taiwan; 5 Li-Yin Technology Co., Ltd., No. 37, Lane 151, Section 1, Zhongxing Road, Wugu District, New Taipei City 241, Taiwan; 6 Department of Mechanical Engineering, National Taipei University of Technology, No. 1, Section 3, Zhongxiao E. Road, Da’an District, Taipei City 106344, Taiwan

**Keywords:** polyetheretherketone, variable rotational speed, rotational friction welding, peak temperature, weld joint

## Abstract

Polyetheretherketone (PEEK) is a promising biomaterial due to its excellent mechanical properties. Most PPEK manufacturing methods include additive manufacturing, injection molding, grinding, pulse laser drilling, or incremental sheet forming. Rotary friction welding (RFW) is a promising bonding technique in many industries. However, very few studies have focused on the RFW of PEEK. Conventionally, the number of revolutions is fixed during the welding process. Remarkably, the rotary friction welding of PEEK polymer rods using an innovative variable rotational speed is investigated in this study. The average bending strength of the welded part using a three-stage transformation rotational speed was enhanced by about 140% compared with a rotational speed of 1000 rpm. The advantage of computer numerical controlled RFW of PEEK using variable rotational speed is a reduced cycle time of RFW. A reduction in cycle time of about 6% can be obtained using the proposed RFW with a three-stage transformation rotational speed. The innovative approach provides low environmental pollution and high energy efficiency and complies with sustainable development goals.

## 1. Introduction

The features of rotary friction welding (RFW) [1] include no electric arc, low energy consumption [2], and low environmental pollution [3]. Therefore, RFW is extensively employed in the joints of many components [4], such as the fabrication of automotive piston rods, shafts, and tubes [5]. RFW is an inertia friction welding used to join two cylindrical components together, providing many advantages, including shorter cycle times, reduced material waste, high joint strength, and the ability to join similar or dissimilar materials.

Iftikhar et al. [6] investigated the friction stir welding friction and stir spot welding of polymer composites and thermoplastic polymers according to medium conditions, tooling conditions, joining materials, and joint configurations. Results showed that the ultimate tensile strength reached 247 MPa, showing a reduction in the material flow gradients. Khalaf et al. [7] studied the heat generation of the parts of the different tools. Results showed that the heat generation in the pins with a triangular shape was more significant than in the pins with a smooth shape. Vidakis et al. [8] focused on travel speed, welding tool pin geometry, and the rotational speed of acrylonitrile butadiene styrene (ABS) parts. It was found that the friction stir welding is a cost-effective process for joining 3D-printed ABS parts. Skowronska et al. [9] studied the structural properties of the weld joints using high-speed friction welding. It was found that a surface hardness exceeding HV 340 can be obtained in a weld joint. Eliseev et al. [10] studied the microstructural evolution in the transfer layer of aluminum alloy welds. It was found that the grain size of incoherent intermetallic particles and the volume fraction decreased towards the center of the layer. Anwar et al. [11] found that the grain size of incoherent intermetallic particles and the volume fraction decreased towards the center of the aluminum alloy welds. Results showed that the minimum grain size can be obtained with a post-weld solution heat treatment.

Polyetheretherketone (PEEK) is a standard implant material that is widely employed in dental implants. PEEK polymer is also widely employed in engineering applications [12]. It is a semicrystalline thermoplastic with excellent chemical and mechanical resistance properties [13]. PEEK has a high melting temperature of about 350 °C [14]. As a result, PEEK is widely used in reinforcing rods. In general, the number of revolutions is constant during the welding process [15], such as 1125 rpm [16], 1350 rpm [17,18], 950 rpm [19], 650 rpm [20], 800 rpm [21], 1600 rpm [22,23], 1000 rpm [24], 1400 rpm [25], 1200 rpm [26], or 900 rpm [27]. This study proposes an innovative method for the RFW of PEEK polymer rods by varying the rotational speed. A thermal camera was used to record the peak temperature in the weld interface during RFW. After RFW, the mechanical properties of the welded parts were investigated by three-point bending and shore A surface hardness tests.

## 2. Experimental Details

**Figure 1:**
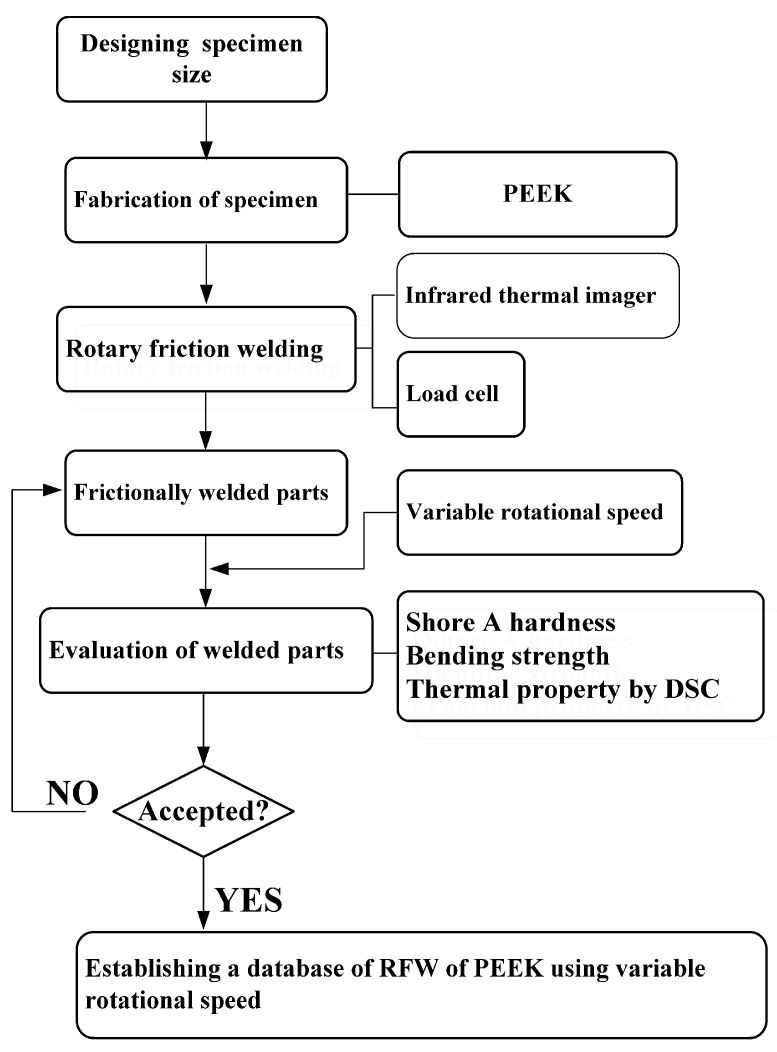
Flowchart of the research process in this study.

**Figure 2:**
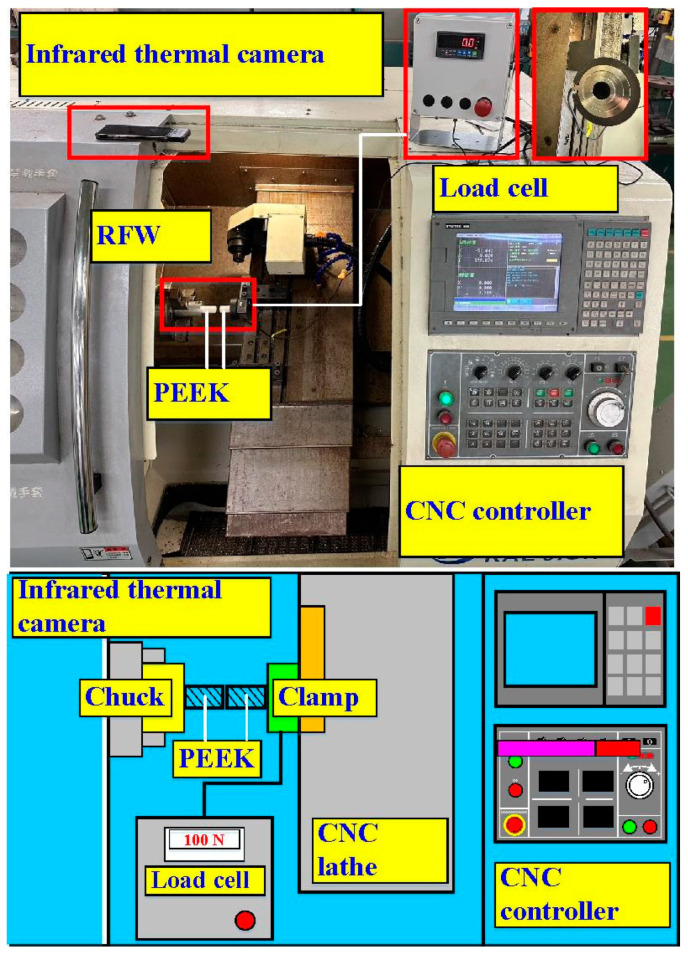
Experimental set-up for RFW of PEEK using variable rotational speed.

**Figure 3:**
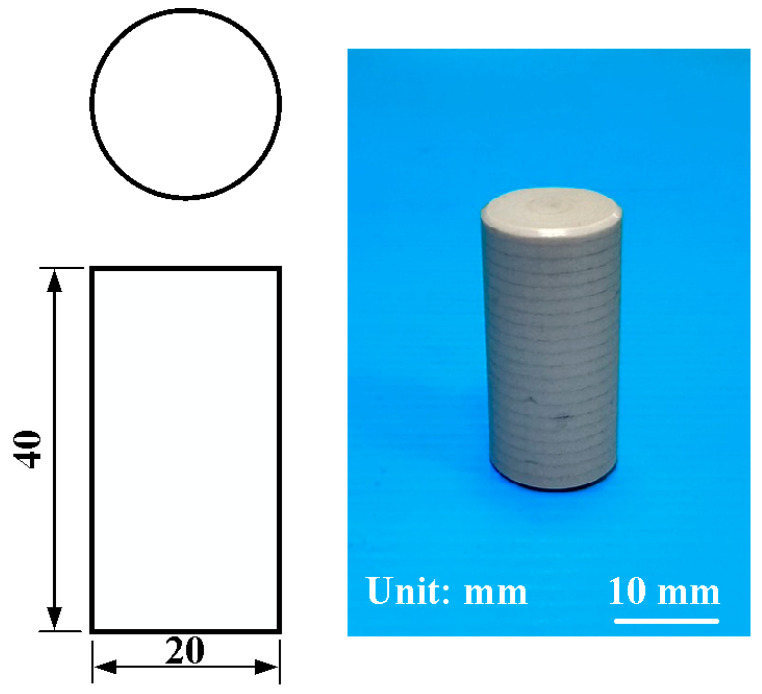
The geometry and size of the RFW specimen.

Figure 1 is the flowchart of the research process. In this study, a computer numerical control turning machine (K-45L, Kae Jiuh, Inc., New Taipei City, Taiwan) was used to join PEEK polymer rods. Figure 2 shows the experimental set-up for RFW of PEEK using variable rotational speed. Initially, one workpiece is fixed with a chuck and is rotated at a constant rotational speed. The other workpiece is firmly held stationary. During RFW, frictional heat is generated at the interface of two workpieces. The workpieces are held under pressure until the weld joint is formed. The temperature of the weld joint was measured during RFW using an infrared camera (BI-TM-F01P, Panrico trading Inc., New Taipei City, Taiwan). A load cell (ARI742, Zhiheng Industrial Co., Inc., New Taipei City, Taiwan) was used to measure forging force during RFW. Figure 3 shows the geometry and size of the RFW specimen. The welding specimen has a round cross-section. The diameter and length are about 20 mm and 40 mm, respectively.

**Figure 4:**
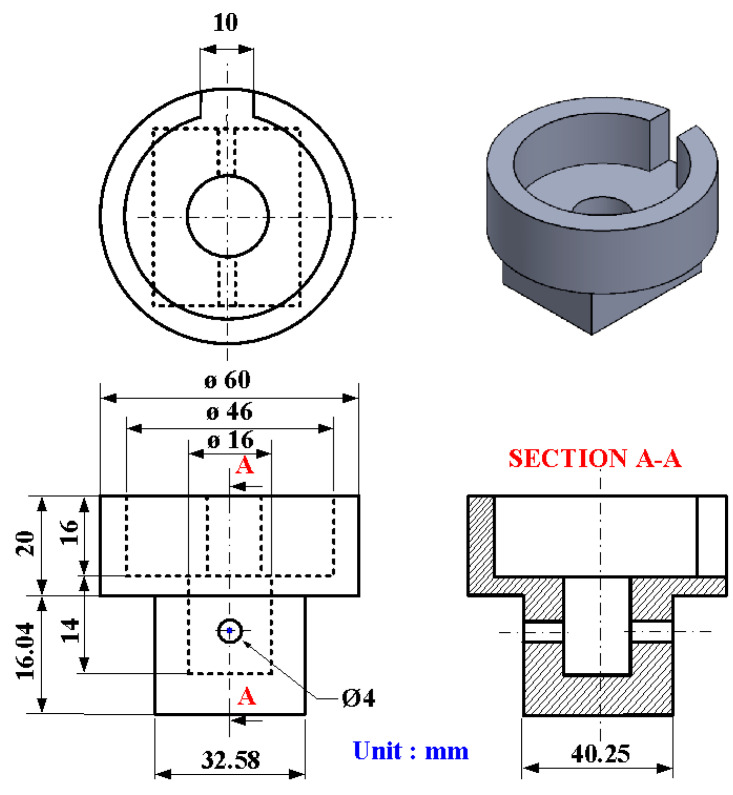
The geometry and size of the jig for the load cell.

**Figure 5:**
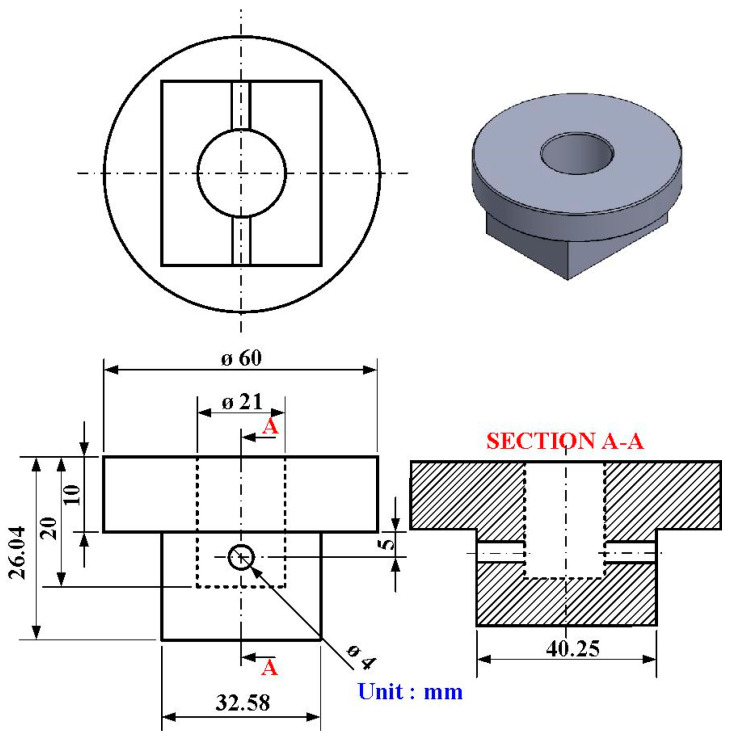
The geometry and size of the jig for the stationary workpiece.

**Figure 6:**
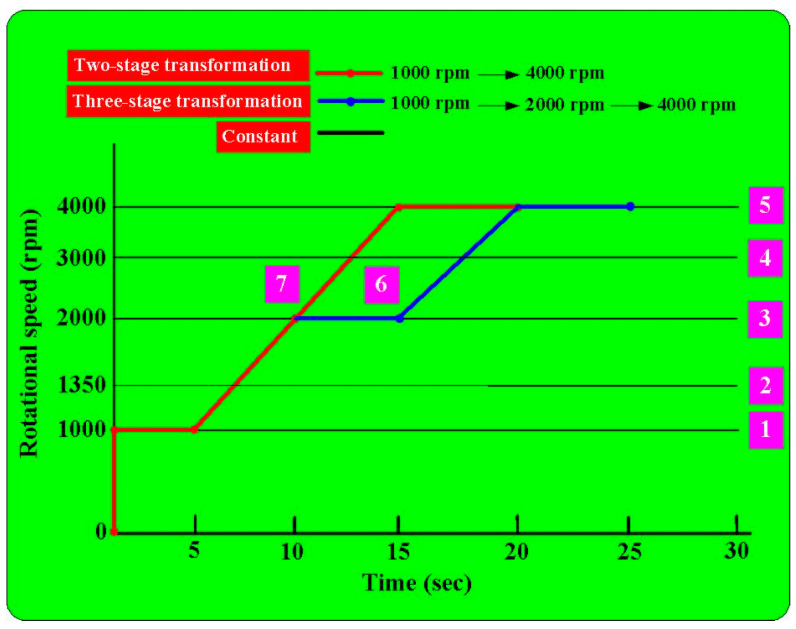
Arrangement of the six rotational speeds of RFW.

**Figure 7:**
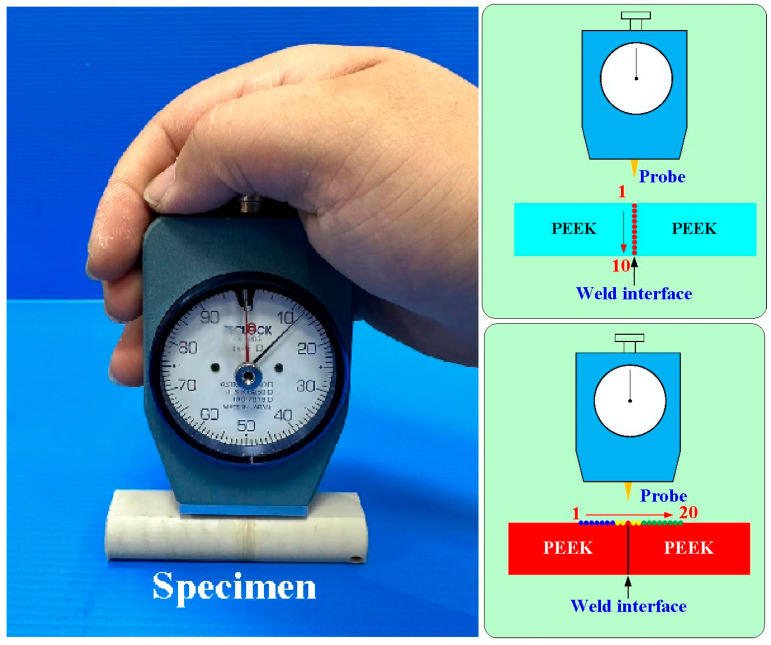
Schematic diagram of the surface hardness measurement location.

Figure 4 shows the geometry and size of the jig for the load cell. The height and width of the jig are about 36.04 mm and 32.58 mm, respectively. The outer and inner diameters are about 46 mm and 16 mm, respectively. Figure 5 shows the geometry and size of the jig for the stationary workpiece. The height and outer diameter are 26.04 mm and 60 mm. Figure 6 shows the arrangement of the six rotational speeds of RFW. As can be seen, there are seven different rotational speeds for RFW. In this study, the rotational friction-welded parts made by the rotational speed of 4000 rpm are the control group, with the number of 5. The experimental groups are 1, 2, 3, 4, 6, and 7. The number of rotating speeds for the numbers 1, 2, 3, 4, and 5 is constant. The rotational speeds are 1000 rpm, 1350 rpm, 2000 rpm, 3000 rpm, and 4000 rpm, respectively. The total RFW time of the two-stage transformation of RFW is 90 s, which includes a friction time of 30 s, a welding time of 30 s, and a cooling time under pressure of 30 s. The number 6 is the two-stage transformation of the rotational speed. The first stage is to accelerate from a standstill to 1000 rpm. The second stage is to accelerate from 1000 rpm to 4000 rpm. The number 7 is the three-stage transformation of the rotational speed. The first stage is to accelerate from a standstill to 1000 rpm. The second stage is to accelerate from 1000 rpm to 2000 rpm. The third stage is to accelerate from 2000 rpm to 4000 rpm. The total RFW time of the two-stage transformation of RFW is 80 s, which includes a friction time of 30 s, a welding time of 20 s, and a cooling time under pressure of 30 s. The total RFW time of the three-stage transformation of RFW is 85 s, which includes a friction time of 30 s, a welding time of 25 s, and a cooling time under pressure of 30 s. Figure 7 shows the schematic diagram of the surface hardness measurement location. There are 10 weld interface measurement points. There are 20 measurement points for the hardness distributions of the welded parts. The shore A surface hardness test (MET-HG-A, SEAT Inc. New Taipei City, Taiwan) and three-point bending test (RH-30, Shimadzu Inc., Kyoto, Japan) were used to assess the weld quality. The optical microscope (OM) (Quick Vision 404, Mitutoyo Inc., Tokyo, Japan) and field-emission scanning electron microscope (FE-SEM) (JEC3000-FC, JEOL Inc., Tokyo, Japan) were used to investigate the microstructure of the weld interface of welded parts. The thermal transitions of the welded parts of RFW using seven different rotational speeds were examined using differential scanning calorimetry (DSC) (STA 409 PC Luxx Simultaneous thermal analyzer, Netzsch-Gerätebau GmbH Inc., Selb, Germany). A mass of 10–15 mg of the welded joint samples was placed in platinum crucibles for the DSC. The specimens were heated at a temperature ranging from 30 °C to 400 °C under the nitrogen gas flow rate of about 25 cc/min [28]. Both the heating rate and cooling rate were 10 °C/min. Finally, a database of RFW of PEEK biopolymer rods using variable rotational speed was established.

## 3. Results and Discussion

**Figure 8:**
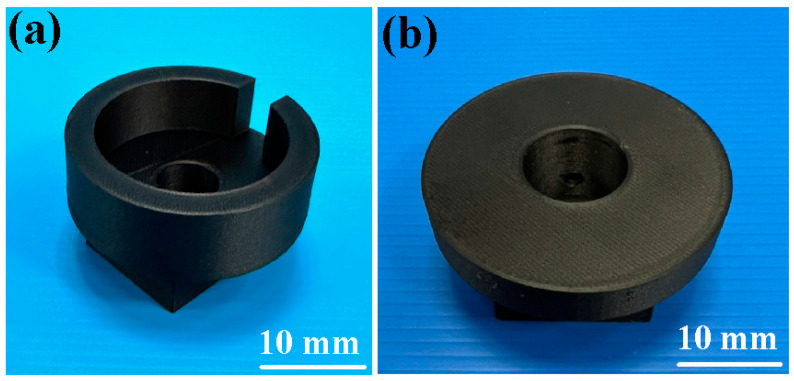
Jigs for (**a**) load cell and (**b**) stationary workpiece of RFW.

**Figure 9:**
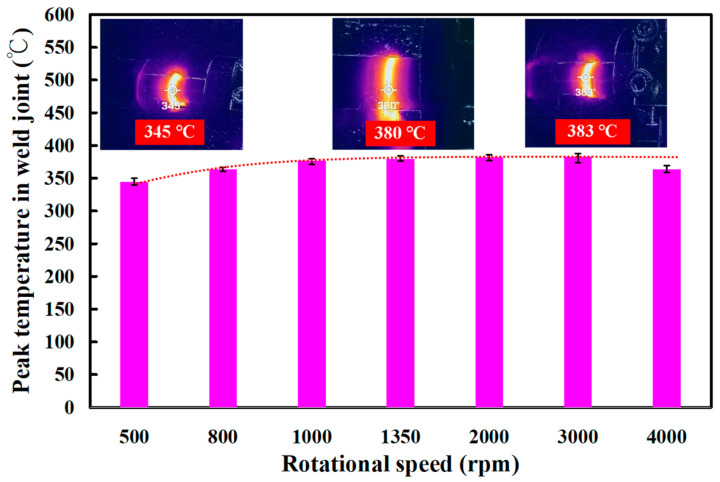
Peak temperature in the weld joint of five different rotational speeds.

**Figure 10:**
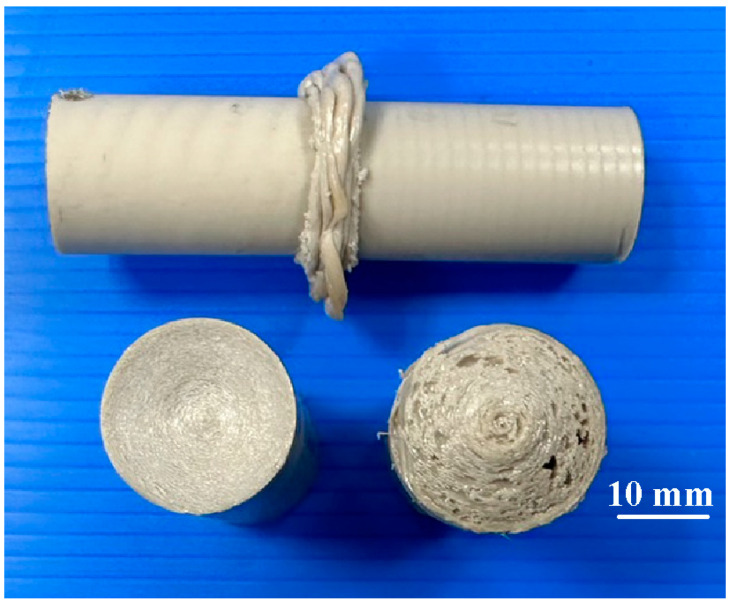
Rotary frictionally welded part with a rotational speed of more than 4000 rpm.

**Figure 11:**
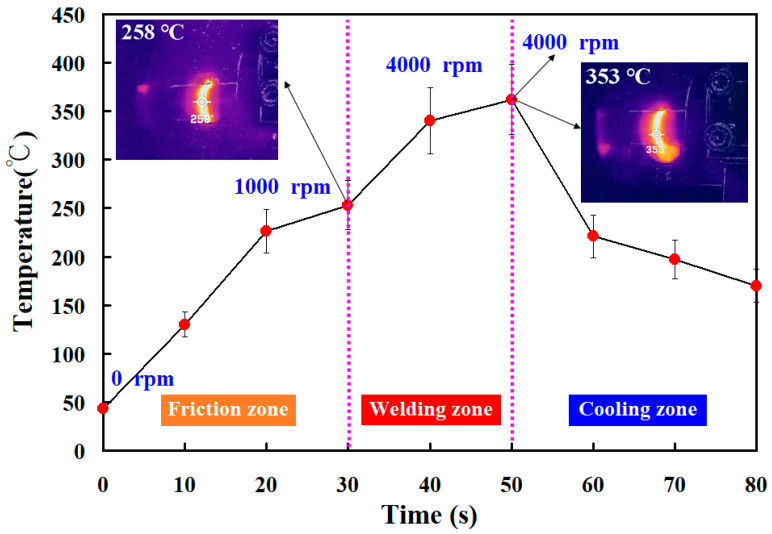
The relationship between weld interface temperature and time for the two-stage transformation of RFW.

**Figure 12:**
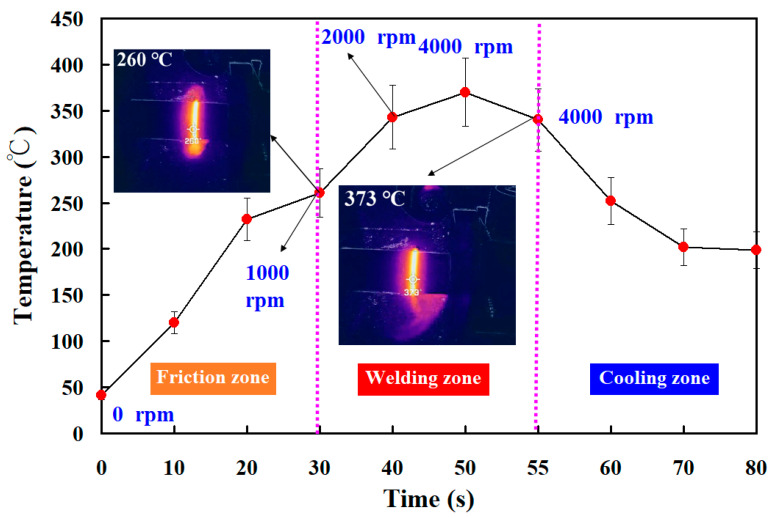
The relationship between weld interface temperature and time for the three-stage transformation of RFW.

Figure 8 shows the jigs for the load cell and stationary workpiece. Two jigs were made with 3D printing technology. In this study, tests were performed on five specimens. Figure 9 shows the peak temperature in the weld joints of five different rotational speeds. It should be noted that the average peak temperature in the weld joint for rotational speeds of 500 rpm and 1000 rpm is lower, which is about 10–30 °C higher than the melting point of PEEK. The average peak temperature in the weld joint for the rotational speed of 1000–4000 rpm exhibits a steady state. The average peak temperature in the weld joint for rotational speeds of 1000 rpm, 1350 rpm, 2000 rpm, 3000 rpm, and 4000 rpm is about 377 °C, 380 °C, 382 °C, 383 °C, and 363 °C, respectively. These results revealed two phenomena. One is that the average peak temperature in the weld joint for rotational speeds of 1000 rpm, 1350 rpm, 2000 rpm, 3000 rpm, and 4000 rpm was about 377 °C, which is about 40 °C higher than the melting point of PEEK [29]. This result is also confirmed by Mir et al. [30] and Wu et al. [31] and occurs because the joined surfaces are heated to melting point during the pressure welding process. The other is that the average peak temperature of the weld bead is slightly lower when the rotational speed of RFW is 4000 rpm. The main reason is that the high rotational speed causes air convection, accelerating the cooling of the peak temperature in the weld joint during RFW. Figure 10 shows the rotary frictionally welded part with a rotational speed of more than 4000 rpm. The results show that the weld bead material has undergone degradation [32,33] and has no joining ability. Therefore, the rotation speed of 1000 rpm is selected as the lowest rotational speed and the rotation speed of 4000 rpm is selected as the highest. Figure 11 shows the relationship between weld interface temperature and time for the two-stage transformation of RFW. The total time of the two-stage transformation of RFW is 80 s. In the friction zone, the weld joint temperature can reach about 258 °C. The weld joint temperature can reach about 353 °C in the welding zone. Figure 12 shows the relationship between weld interface temperature and time for the three-stage transformation of RFW. The total time of the two-stage transformation of RFW is 85 s. In the friction zone, the weld joint temperature can reach about 260 °C. In the welding zone, the weld joint temperature can reach about 373 °C.

**Figure 13:**
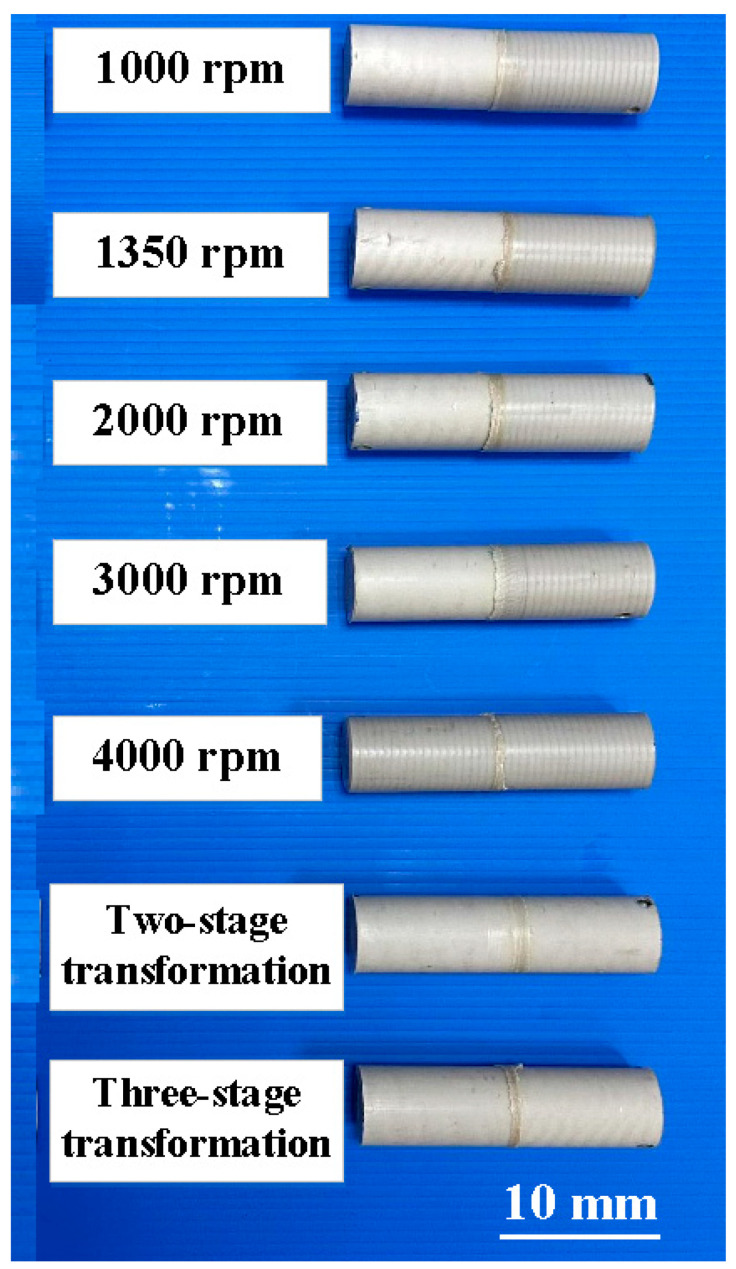
Welded parts of RFW using seven different rotational speeds after removing flash.

In this study, the welding conditions of RFW of PEEK include a burn-off length of RFW of 1 mm; the welding pressure is 0.17 MPa and the feed rate is 6 mm/min. Seven different welding rotational speeds are used for RFW. Figure 13 shows the welded parts of RFW using seven different rotational speeds after removing the flash collar [34]. The height of the flash is about 1.15–5.1 mm.

**Figure 14:**
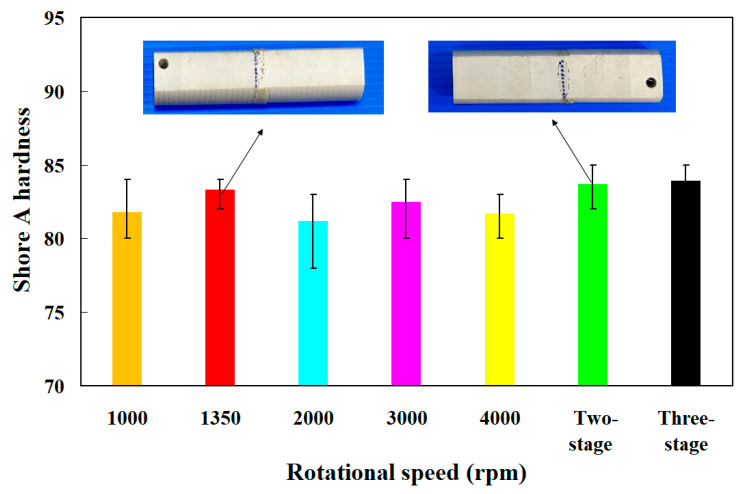
Average surface hardness of the weld interface for seven welded parts.

**Figure 15:**
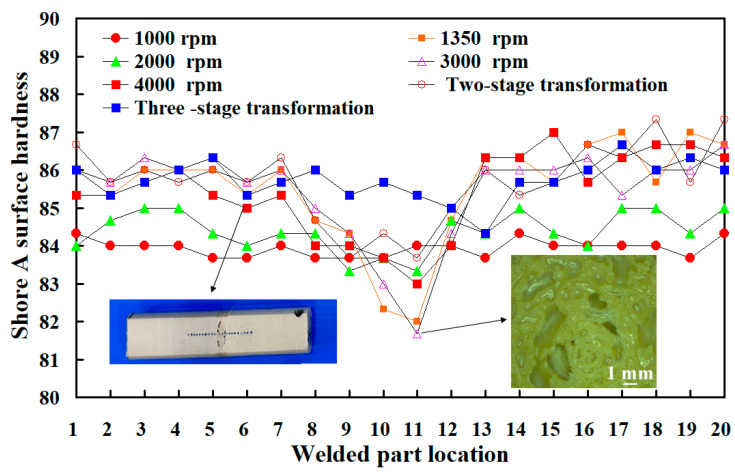
Surface hardness distributions of the seven welded parts.

Figure 14 shows the average surface hardness of the weld interface for seven welded parts. Figure 15 shows the surface hardness distributions of the seven welded parts. The results showed that the average surface hardness of the seven welded parts is about HS 81.8, HS 83.3, HS 81.2, HS 82.5, HS 81.7, HS 83.7, and HS 83.9, respectively. According to these results, two phenomena were observed. One is that the average surface hardness of the weld interface obtained by changing the rotational speed is higher than that of the weld interface obtained by rotating friction welding at a fixed rotational speed. The other is that the average surface hardness of the weld interface is the highest in the three-stage transformation of the rotational speed for RFW, followed by the average surface hardness of the weld interface in the two-stage transformation of the rotational speed for RFW. Microvoids [35] were found in the weld interface of PEEK RFW using the rotational speed of 3000 rpm, resulting in the average surface hardness of the weld interface being lower than that of the base material, PEEK. The reduction rate of surface hardness was about 3.6%. The reason for the microvoids is that the cooling rate of the molten material in the weld interface during RFW is faster than the moving speed of the caused bubbles.

**Figure 16:**
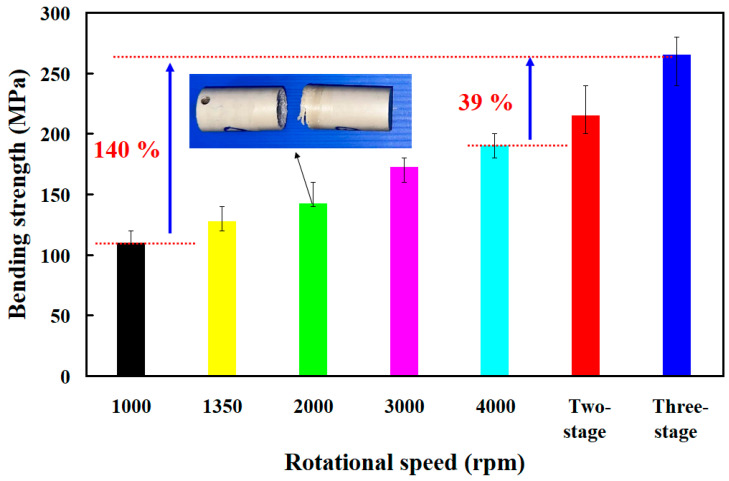
Bending strength of the welded parts of RFW using seven different rotational speeds.

**Figure 17:**
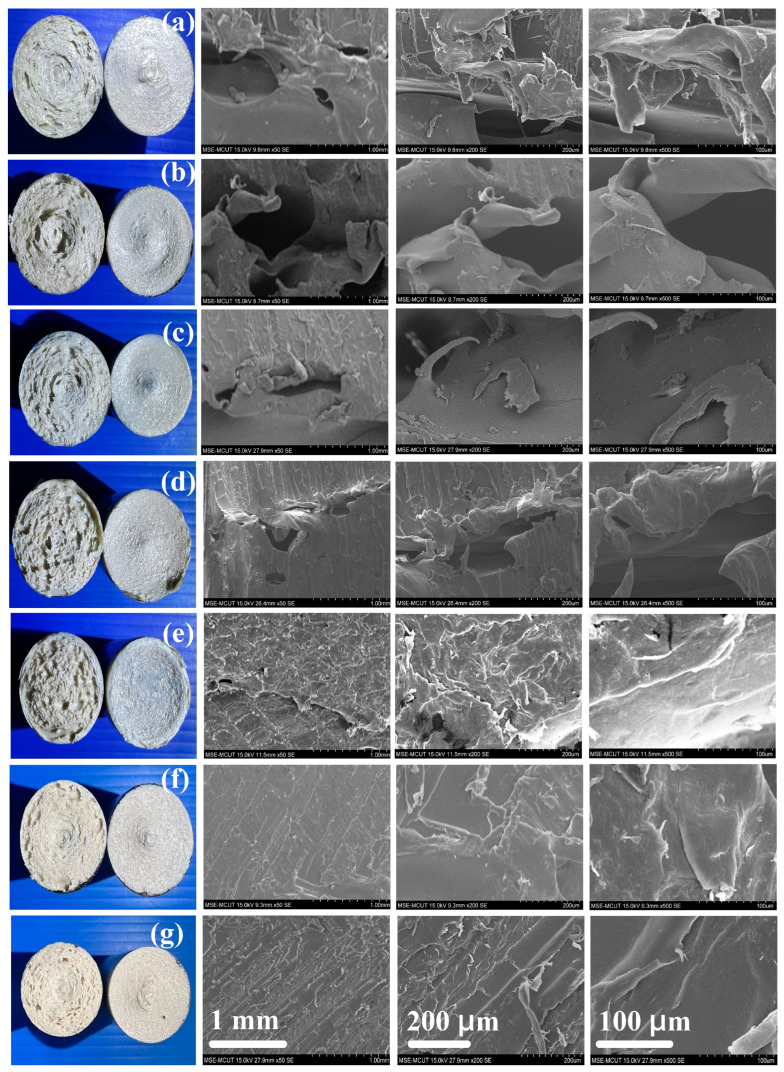
SEM micrographs of fracture surfaces of welded parts of RFW using seven different rotational speeds (**a**) 1000 rpm, (**b**) 1350 rpm, (**c**) 2000 rpm, (**d**) 3000 rpm, (**e**) 4000 rpm, (**f**) two-stage transformation, and (**g**) three-stage transformation.

Figure 16 shows the bending strength of the welded parts of RFW using seven different rotational speeds. The average bending strength of the welded parts using seven different rotational speeds is about 110 MPa, 127.5 MPa, 142.5 MPa, 172.5 MPa, 190 MPa, 215 MPa, and 265 MPa, respectively. According to the above results, this study found four phenomena: (a) the average bending strength of the welded part using variable rotational speed is higher than that of the welded part using fixed rotational speed; (b) The average bending strength of the welded part using three-stage transformation RFW is the highest, followed by the two-stage transformation RFW. It should be noted that the thermal analysis also confirms this result. The heat capacities of the welded parts using three-stage transformation RFW, two-stage transformation RFW, and 1000 rpm are about 0.72 mW/mg, 0.7 mW/mg, and 0.31 mW/mg, respectively. This result shows that the molecular orientation in the weld interface of the welded parts fabricated with three-stage transformation is highest; (c) the average bending strength of the welded part using two-stage transformation rotational speed was enhanced by about 95%, 68%, 50%, 24%, and 13% compared with five fixed rotational speed; and (d) The average bending strength of the welded part using three-stage transformation rotational speed was enhanced by about 140%, 107%, 85%, 53%, and 39% compared with five fixed rotational speed. The total RFW time using fixed rotational speed was 90 s. The total RFW time of the three-stage transformation was 85 s. This study found that the total amount of RFW time saved using a three-stage transformation rotational speed is about 6%. Figure 17 shows the SEM micrographs of fracture surfaces of the welded parts of RFW using seven different rotational speeds. It should be noted that the pores were observed in the fracture surfaces of the welded parts. The reduction in the pores was observed as the rotational speed increased. The pores were fewer for the welded parts fabricated with three-stage transformation rotational speed, and this will help the bending strength of the welded parts.

According to the results described above, this technique can be used for medical applications [36] because PEEK is a standard implant material [37]. According to the literature review, PEEK plastic parts can also be joined by ultrasonic welding (UW) [38,39,40]. However, the significant disadvantage of ultrasonic welding is that it is limited to thinner and smaller components. This study uses a computerized numerical control lathe to join PEEK polymer rods using RFW. The feature of this method is that it is suitable for joining thicker or large PEEK plastic parts. The research results have industrial applicability and practical value because it is a sustainable manufacturing process with low environmental pollution and energy consumption. Therefore, this technology complies with the sustainable development goals 7,9, and 12 [41,42,43]. Fiber laser [44,45,46,47] or carbon dioxide laser [48,49,50] was also suggested to join PEEK polymeric rods. In addition, COMSOL [51] or ANSYS [52] simulation software can be used to predict the maximum interface temperature, total deformation, or equivalent von Mises stress [53] under different rotational speed, axial pressure, friction time, welding time, feed rare, or burn-off length. The tensile testing of the welded parts can also be performed to assess the welding quality. In addition, the effects of the burn-off length on the weld quality of PEEK polymer rods are an exciting research topic. These are interesting research topics and are currently being investigated.

## 4. Conclusions

The main objective of this study was to propose an approach for the RFW of PEEK polymer rods by varying the rotational speed. After FRW, the mechanical properties of the welded parts were examined using a shore A surface hardness test and three-point bending test. The main conclusions from the experimental work in this study are as follows:

1. The average peak temperature in the weld joint for rotational speeds of 1000 rpm, 1350 rpm, 2000 rpm, 3000 rpm, and 4000 rpm was about 377 °C, which is about 40 °C higher than the melting point of PEEK. The average peak temperature of the weld bead is slightly lower when the rotational speed of RFW is 4000 rpm. The main reason is that the high rotational speed causes air convection, accelerating the cooling of the peak temperature in the weld joint during RFW.
2. For the two-stage transformation of RFW, the weld joint temperature can reach about 258 °C in the friction zone and the weld joint temperature can reach about 353 °C in the welding zone. For the two-stage transformation of RFW, the weld joint temperature can reach about 260 °C in the friction zone and the weld joint temperature can reach about 373 °C in the welding zone.
3. The average bending strength of the welded part using three-stage transformation rotational speed was enhanced by about 140%, 107%, 85%, 53%, and 39% compared with five fixed rotational speeds. A reduction in the cycle time of about 6% can be obtained using a three-stage transformation rotational speed.

## Data Availability

Data and materials are available.
